# Comprehensive Analysis of Set-Up Gain of 6-Dimensional Cone-Beam CT Correction Method in Radiotherapy for Head and Neck and Brain Tumors

**DOI:** 10.1155/2022/2964023

**Published:** 2022-10-20

**Authors:** Hoon Sik Choi, Ki Mun Kang, In Bong Ha, Bae Kwon Jeong, Jin Ho Song, Chul Hang Kim, Hojin Jeong

**Affiliations:** ^1^Department of Radiation Oncology, School of Medicine, Gyeongsang National University, Jinju, Republic of Korea; ^2^Department of Radiation Oncology, Gyeongsang National University Changwon Hospital, Changwon, Republic of Korea; ^3^Institute of Health Sciences, Gyeongsang National University, Jinju, Republic of Korea; ^4^Department of Radiation Oncology, Gyeongsang National University Hospital, Jinju, Republic of Korea; ^5^Department of Radiation Oncology, Seoul St. Mary's Hospital, College of Medicine, The Catholic University of Korea, Seoul, Republic of Korea

## Abstract

This study quantitatively analyzed the gain of the six-dimensional (6D) cone-beam CT (CBCT) correction method compared with the conventional set-up method in 60 patients who underwent radiation treatment of head and neck and brain tumors. The correction gain of CBCT was calculated for the translational and rotational motion components separately and in combination to evaluate the individual and overall effects of these motion components. Using a statistical simulation mimicking the actual set-up correction process, the effective gain of periodic CBCT correction during the entire treatment fraction was analyzed by target size and CBCT correction period under two different correction scenarios: translation alone and full 6D corrections. From the analyses performed in this study, the gain of CBCT correction was quantitatively determined for each situation, and the appropriate CBCT correction strategy was suggested based on treatment purpose and target size.

## 1. Introduction

The goal of radiotherapy (RT) is to deliver a sublethal dose to cancer cells while protecting surrounding healthy tissues. To achieve this goal, the accuracy of radiation targeting must be explicitly guaranteed. The traditional set-up method for RT [1] consists of taking two orthogonal planar images of a patient in a separate simulation room, matching the live simulation images with the patient's planned images, and marking the set-up fiducial lines on the patient's body. The patient is then moved to the treatment room and positioned so that the set-up fiducial lines coincide with the laser crosshairs prealigned to the center of treatment. Although this method has long been validated and is likely to be sufficiently effective in general situations, it has several limitations, including difficulties reflecting anatomical changes that occur during treatment and the need to correct for rotational misalignment.

Recent advances in imaging technology have resulted in the development of various six-dimensional (6D) image guidance methods [2], such as cone-beam computed tomography (CBCT) [3–5], magnetic resonance imaging (MRI) [6, 7], and stereovision surface imaging [8, 9], to overcome these limitations. The CBCT method is regarded as the standard for patient set-up in RT [3, 7, 9] because it provides not only the sophisticated three-dimensional (3D) images necessary for set-up but also intuitive information directly comparable with treatment planning CT images. Other image-guided methods, such as MRI [6, 7] and stereovision images [8, 9], have little relation to CT images and have not been widely adopted in clinical practice.

The most crucial drawback associated with the use of CBCT in RT is the inevitable increased exposure to radiation for nontherapeutic purposes. Large cohort studies have reported that CT scans increase cancer risks [10–12], with studies in the United Kingdom [11] and Australia [12] showing that CT scans in children increase their lifetime risk of cancer incidence by approximately 24%. CBCT scans are likely associated with similar risk, because the mechanism and X-ray energy bands used for CBCT scanning are basically the same as those used for conventional CT [4].

In performing CBCT, it is therefore very important to balance the gain from set-up accuracy with the loss due to increased cancer risk. Many numbers of studies have been conducted to investigate CBCT efficacy in radiation therapy. These include analysis of translation and rotation errors in patient positioning [13–19], the accuracy and margin reduction effect of CBCT [14, 20, 21], the dosimetry related to the patient set-up error [13], the effects and comparisons of 6D image guidance methods [22, 23], and suggestion of optimal margins for CBCT-based radiation treatment [19, 21]. However, none of these studies considered the coupled effect of translation and rotation errors and presented an appropriate correction frequency.

As part of a preliminary study to determine the appropriate balance for CBCT usage in RT, the actual set-up errors occurring during RT were quantified in detail by simultaneously evaluating rotation and translation errors and by determining the relationships between gains associated with 6D CBCT correction and correction frequency. CBCT correction efficiency varies from site to site [18, 19, 21]. Because head and neck (H&N) tumors and brain tumors are less affected by factors other than external set-up error, such as internal movement and tumor deformation, the present study assessed CBCT correction efficiency in patients undergoing RT for H&N and brain tumors.

## 2. Methods and Materials

### 2.1. Data Selection

Target volume coordinates and daily set-up position data were collected to verify the effectiveness of the CBCT-based 6D correction method. These data were obtained from the treatment records of patients undergoing RT for H&N and brain tumors at our institution. Patients were included if (i) the target volumes of their tumors were localized only within H&N and brain regions, (ii) their set-up positions were periodically checked by CBCT, and (iii) they were immobilized in a supine position using thermoplastic head masks. Patients were excluded if (i) their treatments were replanned during the course of treatment due to significant changes in tumor morphology; (ii) they were positioned by other image guidance methods, such as MV or KV portal imaging, in combination with CBCT; (iii) they required additional immobilization tools, e.g., tongue bite for oral cavity treatment; or (iv) their performance status was too poor to allow a normal set-up process.

The 60 patients selected for the present study included 30 with H&N tumors, including seven with treatment sites involving the nasopharynx to the paranasal sinuses, 10 with treatment sites involving the oropharynx and salivary glands, six with treatment sites involving the hypopharynx or larynx, and seven with treatment sites in other areas of the neck. The other 30 patients included 15 with primary and 15 with metastatic brain tumors.

The target volumes for these patients were determined by qualified radiotherapy oncologists with the addition of planning margins ranging from 3 to 10 mm, depending on the treatment purpose. Analysis in patients with multiple targets focused on the largest target.

### 2.2. Patient Set-Up Procedure

All patients were initially positioned via the conventional laser set-up procedure, in which patients were manually aligned so that the set-up fiducial lines marked on the immobilization head mask matched the laser crosslines prealigned to the isocenter of our LINAC system (TrueBeam 2.0, Varian Medical Systems, Palo Alto, CA). Patients were subsequently positioned by the CBCT image guidance set-up procedure, in which patients underwent CBCT scanning in the laser set-up position and were repositioned, if necessary, by 6D CBCT guidance. The laser set-up procedure was performed daily from the beginning to the end of the treatment, whereas the CBCT set-up procedure was performed prior to the first treatment and generally once per week thereafter. However, if the set-up difference was >2 mm in any axis, the CBCT set-up was performed on at least three consecutive days; if the difference persisted, the set-up fiducial lines were refined to the CBCT corrected position.

The positions of the laser and CBCT set-ups for each patient were logged in real time into the ARIA record and verification system (Varian Medical Systems). These records were extracted and analyzed in the study.

### 2.3. Individual Target Error Calculation

The target position error was calculated based on the assumption that the CBCT set-up was sufficiently accurate, whereas the laser set-up could be erroneous. Based on this assumption, the target position error (Δ) was defined as the difference in position of the laser set-up (*P*_La_) relative to that of the CBCT (*P*_CBCT_) set-up, as expressed in the following equation:

(1) $$\begin{array}{c} \Delta =P_{\text{La}}-P_{\text{CBCT}}. \end{array}$$

This defined set-up error does not represent the absolute error, but the relative error to that of the CBCT set-up, regarded as the standard.

The set-up position and error were usually determined using a 6D coordinate system, consisting of three translational axes, anterior-to-posterior (AP), left-to-right (LR), and superior-to-inferior (SI), expressed in distance units, and three rotational axes, yaw, pitch, and roll, expressed in angular units. Translational (Δ_T_) and rotational (Δ_R_) error components were analyzed separately and in combination for each patient. Translational error was defined as an error that uniformly shifts the target volume, with the magnitude of the shift being equal for all the points in the target volume; translational errors were therefore easily calculated from the difference in Cartesian coordinates between the laser and CBCT set-up positions. In contrast, target movement induced by rotational error differed from point to point in the target volume, depending on the distance of the point from the center of rotation. The coordinates of the target point relative to the center of rotation were therefore also required to calculate the sweep distance resulting from the rotation, which was calculated using a Euler rotational transformation matrix [24]:

(2) $$\begin{array}{c} \left(\begin{array}{c} x_{r}\\ y_{r}\\ z_{r} \end{array}\right)=\left(\begin{array}{ccc} 1 & 0 & 0\\ 0 & \cos\left(\theta_{x}\right) & -\sin\left(\theta_{x}\right)\\ 0 & \sin\left(\theta_{x}\right) & \cos\left(\theta_{x}\right) \end{array}\right)\cdot \left(\begin{array}{ccc} \cos\left(\theta_{y}\right) & 0 & \sin\left(\theta_{y}\right)\\ 0 & 1 & 0\\ -\sin\left(\theta_{y}\right) & 0 & \cos\left(\theta_{y}\right) \end{array}\right)\cdot \left(\begin{array}{ccc} \cos\left(\theta_{z}\right) & -\sin\left(\theta_{z}\right) & 0\\ \sin\left(\theta_{z}\right) & \cos\left(\theta_{z}\right) & 0\\ 0 & 0 & 1 \end{array}\right)\cdot \left(\begin{array}{c} x_{0}\\ y_{0}\\ z_{0} \end{array}\right). \end{array}$$

This matrix computes the rotated position (*x*_*r*_, *y*_*r*_, *z*_*r*_) of a target point from the initial position (*x*_0_, *y*_0_, *z*_0_) by the rotational motion around the isocenter (*x*_*c*_, *y*_*c*_, *z*_*c*_) with the rotating angles (*θ*_*x*_, *θ*_*y*_, *θ*_*z*_) along the *x*-, *y*-, and *z*-axes, respectively. The rotation center was set at the center of mass (COM) of the target volume for each patient, as practiced in general radiation treatment. Although the rotation-induced target motion differed from point to point, the rotation-induced target motion in the present study was defined as the maximum shift of all target points.

The overall magnitude of target motion, due to both translation and rotation errors, was calculated by the root-mean-square sum as

(3) $$\begin{array}{c} ∆=\sqrt{{\left(x_{r}+x_{t}-x_{0}\right)}^{2}+{\left(y_{r}+y_{t}-y_{0}\right)}^{2}+{\left(z_{r}+z_{t}-x_{0}\right)}^{2}}, \end{array}$$

with the rotational (*i*_*r*_) and translational (*i*_*t*_) shifted positions along the *i*-axis.

**Figure 1:**
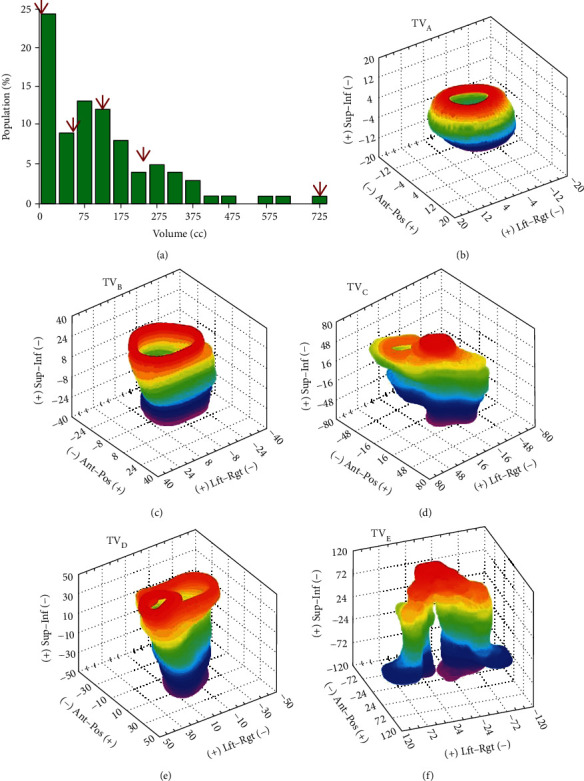
(a) Differential histogram of target volume sizes in 60 selected patients, with volume sizes corresponding to quartiles 0 to 4 of the population indicated by arrows. Five specific targets with volume sizes corresponding to these quartiles were chosen for the present analysis and displayed in (b–f) in the order of volume size, ranging from the smallest (b, TV_A_) to the largest (f, TV_E_). See Table 1 for a detailed description of these five specific targets.

The individual effects of set-up errors on target shift should be evaluated using individual target coordinates, but here, we applied the Euler matrix calculation to only five specific target volumes shown in Figure 1 for computational efficiency.

### 2.4. Effective Error over All Treatment Fractions

In actual RT, set-up error varies from day to day over the entire treatment fractions. To effectively quantify these variations, the effective deviation of target volume (Δ_eff_) was defined based on van Herk et al.'s formula [25] which allows the optimal planning margin to be expressed, relative to preparation (*Σ*) and execution (*σ*) errors, as 2.5*Σ* + 0.7*σ*. This formula suggested that execution errors had less impact than preparation errors when both types of errors occurred during the course of treatment, by a factor of 0.7/2.5 [26, 27]. Thus, Δ_eff_ was defined as

(4) $$\begin{array}{c} \Delta_{\text{eff}}=\Delta_{\text{avg}}+\left(\frac{0.7}{2.5}\right)\cdot \Delta_{\text{std}}, \end{array}$$

where Δ_avg_ and Δ_std_ are the average and standard deviation of daily set-up variations over all treatment fractions, respectively, and may correspond to the preparation (*Σ*) and execution (*σ*) errors in van Herk et al.'s formula [25], respectively.

### 2.5. Effective Gain of CBCT Correction

**Figure 2:**
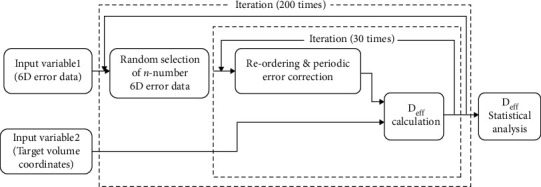
Workflow for the iterative calculation of effective target error (*D*_eff_). The 6D error dataset and target volume coordinates were the input variables, with *D*_eff_ calculated in the Euler rotation matrix equation (Equation (1)).

The gain of periodic CBCT correction compared with the conventional laser set-up was investigated by statistical simulations that mimicked actual CBCT correction procedures. The workflow of the simulation is illustrated schematically in Figure 2.

First, *n*-numbers of the 6D error dataset were randomly constructed from the 358 actual individual datasets collected for this study, which represented the daily set-up errors throughout the *n*-fractionated treatments. Second, among the *n* selected data points in the data array, every *m*th data point from the first was corrected to zero to mimic the periodic CBCT set-up correction for every *m*th fraction. At this stage, two different types of correction were considered: full 6D correction, fully correcting for both translational and rotational errors, and translation-only error correction with no correction for rotation error. Third, the effective target error (Δ_eff_) was calculated by applying the 6D error datasets and target coordinates individually to the Euler transformation matrix as expressed in Equation (2).

The above simulation was iterated 300 times by reconstructing the 6D error dataset and 20 times by random reordering of the dataset, resulting in a total of 6000 iterative calculations for each target volume. The simulation was also repeated for several CBCT correction periods of *m* (*m* = 1 to 10, 15, and 30) with the number of fractions (*n*) set at the multiple of *m* closest to 30. That is, if *m* was 1, 2, 3, 5, 6, 10, 15, or 30, then *n* would be 30; if *m* was 4 or 7, then *n* would be 28; and if *m* was 8, then *n* would be 32.

### 2.6. Ethics Statement

The present study was reviewed and approved by the Institutional Review Board of the Gyeongsang National University Changwon Hospital (approval No. 2022-01-018).

## 3. Results

### 3.1. Set-Up Error Statistics

Table 2 summarizes the statistics of set-up errors measured in the present study. The means ± standard deviations (SDs) of the errors detected in the translational axes were 1.4 ± 1.3 mm (AP), 1.3 ± 1.2 mm (LR), and 1.5 ± 1.4 mm (SI), and the mean ± SD of errors in the rotational axes were 0.6 ± 0.5° (yaw), 0.5 ± 0.5° (pitch), and 0.6 ± 0.6° (roll). The mean ± SD root-mean-square (RMS) sums for the translational and rotational errors were 2.9 ± 1.7 mm and 1.2 ± 0.7°, respectively, comparable to those previously reported [14–17]. The 90th percentile errors along the AP, LR, and SI translational axes were 3.6 mm, 3.4 mm, and 4.2 mm, respectively, resulting in an overall RMS of 5.0 mm. The 90% rotation errors in yaw, pitch, and roll were 1.2° each, with an overall RMS of 2.0°.

### 3.2. Target Volume Statistics

Figure 1(a) shows a differential histogram of the statistical distribution of target volume sizes in the 60 selected patients. Of all 60 target volumes, five specific targets, with volumes equally spaced at 25% intervals, starting from the minimum, were specifically chosen for further analyses. These five targets, called TV_A_ to TV_E_, were 3.0, 45.9, 126.7, 239.7, and 798.2 cc in volume, respectively. The detailed characteristics of these five target volumes are shown in Figures 1(b)–1(f) and summarized in Table 1.

### 3.3. Target Position Error Caused by Individual Set-Up Error

**Figure 3:**
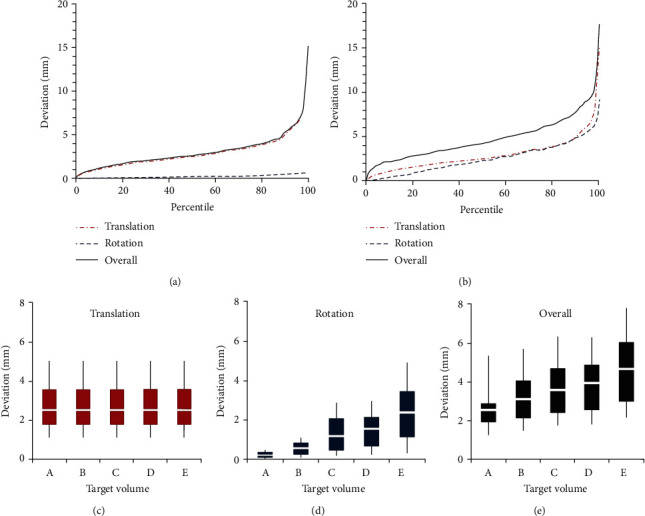
Percent target errors resulting from individual daily set-up error for (a) the smallest (TV_A_) and (b) the largest (TV_E_) target volumes. (c–e) Box and whisker plots for (c) translational, (d) rotational, and (e) overall target errors for the five specific target volumes shown in Figures 1(b)–1(f). Boxes indicate the 25th to 75th percentiles, whiskers indicate the 10th to 90th percentiles, and thicker solid lines indicate the median (50th percentile) of the ranges of target errors.

The target position error caused by individual set-up mismatch was calculated by individually applying the set-up error data to the Euler matrix expressed as Equation (2), using the five selected target volumes seen in Figures 1(b)–1(f). The results calculated for the smallest (TV_A_) and largest (TV_E_) targets are plotted in detail in Figures 3(a) and 3(b), respectively, as a function of percentile distribution. The results for translation, rotation, and overall (translation+rotation) errors from all five targets are displayed as box-and-whisker plots in Figures 3(c)–3(d), respectively. The results showed that the target movements caused by translational errors were independent of tumor size as seen in Figure 3(c), whereas the target movements caused by rotational errors were proportional to target size as seen in Figure 3(d). The 90th percentile of translational movement or the margins required to cover 90% of translational target motions were equal to all the targets at 5.0 mm (Figure 3(c)). In contrast, the 90th percentile of target motion induced by rotational errors was only 0.4 mm for the smallest target (TV_A_) but increased with target size to 4.9 mm for the largest target (TV_E_) (Figure 3(d)). The overall 6D target motion resulting from both translational and rotational errors therefore also increased with target size, on the basis of the 90th percentile, from 5.3 mm for TV_A_ to 7.8 mm for TV_E_, as shown in Figure 3(e).

### 3.4. Effective Target Error over All Fractions

**Figure 4:**
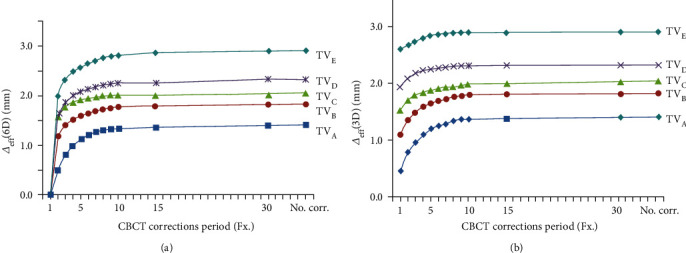
Effective target errors (Δ_eff_) as a function of CBCT correction period calculated for (a) full 6D error correction and (b) 3D-only error correction scenarios.

The fluctuation in target deviation over the entire course of treatment was quantified based on Δ_eff_ defined by Equation (4) using the five selected target volumes seen in Figures 1(b)–1(f). These calculations were performed in full 6D and translation-only (or 3D-only) correction scenarios, with the results plotted in Figures 4(a) and 4(b), respectively, as a function of the CBCT correction period.

The results showed three important features regarding CBCT correction. First, *D*_eff_ was significantly smaller than the target error caused by individual set-up errors (Δ). Assuming no CBCT correction in both the correction scenarios, the 90th percentile of *D*_eff_ was dependent on target size, ranging from 1.4 mm for TV_A_ to 2.9 mm for TV_E_ in both the scenarios (Figures 4(a) and 4(b)), and was more than 50% lower than the 90th percentile of individual target error (Δ), which ranged from 5.3 mm for TV_A_ to 7.8 mm for TV_E_ as shown in Figures 3(a) and 3(b), respectively.

Second, Δ_eff_ obtained using the full 6D correction scenario was subsequently reduced by applying more frequent CBCT corrections (see Figure 4(a)); thirdly, Δ_eff_ obtained with the 3D-only correction scenario had a relatively smaller change in response to the frequency of CBCT corrections, leaving a residual error even after applying the correction to every fractionation schedule. The residual errors in the 3D-only correction scenario ranged from 0.5 mm for TV_A_ to 2.6 mm for TV_E_, with the residual errors for TV_B_, TV_C_, and TV_D_ being intermediate (see Figure 4(b)).

## 4. Discussion and Conclusions

The CBCT-based image-guided set-up method has many advantages over the conventional set-up method, including full 6D patient alignment and visibility as well as correctability in response to daily anatomic changes. In contrast, the exposure of patients to extra radiation in addition to that required for therapeutic purposes is an unavoidable disadvantage of the CBCT method. Because optimizing CBCT is essential in RT, it is necessary to quantitatively determine the efficacy of CBCT.

Although the efficacy of CBCT differs by treatment site [18, 19, 21], the present study assessed the effects of CBCT on brain and H&N tumors because internal tumor motion at these sites is relatively small, making it easier to analyze the efficacy of CBCT at these sites.

The statistics of translational and rotational errors detected by 6D CBCT were first analyzed separately. Translational errors occurring in actual treatment are generally regarded as acceptable if they fall within the commonly used margin range of ~5 mm. The 90th percentile of RMS distance for translational error was estimated to be 5.0 mm (upper whisker in Figure 3(a)), indicating that 90% of all translational set-up errors could be safely compensated for by adding 5 mm planning margins. The magnitude of rotation error, expressed as the median RMS, was 1.13°, with the 90th percentile being 2.03°. This magnitude was also deemed acceptable, as mechanical rotational errors < 2° for LINAC are generally regarded as acceptable [28].

Combined analysis, in which the effects of translational and rotational errors were simultaneously analyzed, found that the overall set-up error could substantially exceed the tolerance limit, even if both types of errors were within the tolerable range. This problem mainly appeared in large-size targets because rotation-induced motion distance increased with target size. For example, as can be found in Figures 3(c) and 3(d), the 90th percentile of the rotational set-up error (Δ_R_) was much smaller than the 90th percentile of the translational set-up error (Δ_T_) for the smallest target volume (TV_A_; 0.05 mm vs. 5.0 mm), but the two were similar for the largest target volume (TV_E_; 4.9 mm vs. 5.0 mm). This result strongly suggests the need for special care to minimize rotation set-up errors when treating large-size tumors. Because rotational errors cannot be fully corrected with the conventional alignment method, these results also demonstrate the need for 6D alignment methods, such as CBCT, in treating large-sized targets.

This study quantified the magnitude of movement of the target volume during the entire course of treatment, as well as the ability of periodic CBCT correction to reduce movement. The effective target error was found to be relatively small compared with the range of margins currently used in RT regimens (3-5 mm). In the absence of CBCT correction (the rightmost points in Figures 4(a) and 4(b)), the 90th percentile of the effective target error (Δ_eff_) was only 1.4 mm for the smallest target (TV_A_) and did not exceed 2.9 mm for the largest target (TV_E_), despite the tendency of the error to increase with target size. This is basically because positional errors are relatively small in H&N and brain tumors and were averaged over multiple fractionated courses of treatment.

Despite the effective target error being small, the patient set-up accuracy must be checked periodically because of the potential appearance of an error that exceeds the tolerance limit. In the present study, errors exceeding 5 mm tolerance occurred in 11-38% of individual errors (Δ) and in 0~1% of overall effective errors (Δ_eff_), depending on the target size. The effectiveness of periodic set-up correction for CBCT imaging was examined under two different scenarios: 3D-only correction and full 6D correction. The former represents the conventional method of correction, omitting corrections for rotational errors, whereas the latter corresponds to an advanced image-guided method that includes a correction for rotational errors, such as CBCT.

In 3D-only correction, the effective error was not much changed within 1 mm, as can be seen in Figure 4(b), with a certain level of residual error remaining regardless of the frequency of CBCT correction. This result suggests that 3D-only or translational-only correction is not very effective and is largely limited to attaining sufficient precision in patient set-up procedures. In contrast, full 6D correction resulted in a clear decrease in effective error with CBCT correction (see Figure 4(a)), suggesting that more frequent CBCT correction will provide greater accuracy in patient set-up.

These findings provide practical information for applying the 6D CBCT correction method in RT of patients with brain and H&N tumors. Because the effect of rotational error depends on target size, target size should be considered when choosing the 6D correction cycle. In particular, in treating large tumors with long axes > 10 cm, 6D CBCT correction should be regarded as mandatory, at least for the first treatment and at intervals thereafter. Periodic 6D correction is also recommended for precision RT, such as stereotactic radiosurgery in patients with smaller-sized tumors and radiotherapy in patients with tumors very close to critical organs because only very small errors are tolerated in these kinds of treatments, but 3D-only correction likely would result in nonnegligible residual errors. However, 6D CBCT correction may not be clearly better than the conventional 3D method in patients undergoing general treatment for H&N and brain tumors of common size with conventional margins. A 3D set-up method, with relatively low-level exposure to radiation, or a CBCT method with relatively long intervals between corrections may optimize both patient efficacy and safety. The present study indicates that a CBCT correction every 5-7 fractions, providing an accuracy similar to the 3D-only correction scenario, would be sufficient for general treatment.

The present study had several limitations. First, patient set-up error was regarded as only a random error, as the set-up error dataset was randomly selected from data recorded for different patients. In practice, however, systematic error biased in a specific direction may occur in individual patients due to various reasons, such as the mismarking of set-up baselines and patient habits. Second, this study did not analyze extracranial sites, such as the thorax, abdominal, and pelvis, in which CBCT guidance is more necessary due to the greater deformability and movability of tumors at these sites [2, 18, 19, 21]. Another limitation was that the error caused by the CBCT itself was not considered in the study, although there is some uncertainty depending on the correction algorithm [29]. Nevertheless, because CBCT has been reported to be more accurate than other methods used in practice [3, 7, 9], the methodology used in the present study was regarded as practically meaningful.

In conclusion, this study quantified set-up errors for RT of intracranial H&N and brain tumors by simultaneously considering both translational and rotational movements and investigated the effectiveness of the 6D CBCT correction method. In general situations, the 6D correction method did not have a distinct advantage over the conventional 3D correction method. However, the 6D correction method was advantageous in certain situations, such as RT for large tumors, as rotational errors can cause large-scale displacement, and in precision RT, in which even small residual errors may be crucial. The use and frequency of CBCT correction should be determined by carefully considering tumor size, planning margins, and the purpose of treatment.

## Figures and Tables

**Table 1:** The dimensions of the five specific target volumes seen in Figures 1(b)–1(f). Shown are the target volumes, the maximal range from the center of the mass (COM) along each translational axis, and the maximal radial distance from the COM.

Targets	TV_A_	TV_B_	TV_C_	TV_D_	TV_E_
Volume (cc)	3.00	45.9	126.7	239.7	789.2
Range (mm)					
LR^a^	11.8-11.1	24.3-22.8	28.5-29.8	42.7-35.0	115.4-99.3
SI^b^	10.2-11.1	17.4-21.9	28.2-31.9	51.5-70.3	70.8-52.0
AP^c^	5.9-6.1	21.4-23.6	43.9-74.1	63.3-62.7	103.1-97.9
Max.^d^ radius (mm)	12.1	31.0	78.3	96.1	134.9

^a^LR: left-to-right; ^b^SI: superior-to-inferior; ^c^AP: anterior-to-posterior; ^d^Max.: maximum.

**Table 2:** The statistics of set-up error measured from 358 actual patient data.

Axis	Translation (mm)				Rotation (°)			
	AP^a^	LR^b^	SI^c^	RMS^d^	Yaw	Pitch	Roll	RMS^d^
Max.^e^	8.60	10.40	8.00	12.42	2.60	2.50	2.70	3.54
Mean	1.43	1.27	1.53	2.85	0.55	0.52	0.64	1.18
SD^f^	1.19	1.25	1.42	1.70	0.52	0.49	0.60	0.68
Median	1.10	1.00	1.20	2.48	0.40	0.40	0.50	1.12
90%^g^	3.60	3.44	4.22	6.07	1.20	1.20	1.24	2.03

^a^AP: anterior-to-posterior; ^b^LR: left-to-right; ^c^SI: superior-to-inferior; ^d^RMS: root-mean-square sum; ^e^Max.: maximum; ^f^SD: standard deviation; ^g^90%: 90 percentile.

## Data Availability

The data used to support the findings of this study are available from the corresponding author upon request.
